# Very Early‐Onset Behavioral Variant Frontotemporal Dementia: A Case Report

**DOI:** 10.1002/alz70857_104327

**Published:** 2025-12-25

**Authors:** Veronica Perez, Marcelo Jobet, Pablo Toro, Rodrigo A Santibanez

**Affiliations:** ^1^ Neurology Department, Faculty of Medicine, Pontificia Universidad Católica de Chile, Santiago, Chile; ^2^ Memory Clinic, Neurology Service, Complejo Asistencial Doctor Sótero del Río, Santiago, Chile; ^3^ Department of Psychiatry, Faculty of Medicine, Pontificia Universidad Católica de Chile, Santiago, Chile

## Abstract

**Background:**

Frontotemporal dementia (FTD) is a syndrome with clinically and pathologically heterogeneous manifestations. Progressive personality changes, behavioral disturbances, neuropsychiatric symptoms, and cognitive impairment are the hallmarks of this disease. Behavioral variant FTD (bvFTD) is the most prevalent variant, with a common onset between 45 and 64 years old. Although there is no consensus, a very early onset presentation is usually considered when symptoms arise before 30 years old. We present a case report of very early onset bvFTD in Chile

**Method:**

A 23‐year‐old male developed progressive apathy, anxiety, stereotyped behaviors, and social withdrawal. A psychiatric condition was initially suspected and treated accordingly. After four months, patient began exhibiting hypersexualization, disinhibition, and hyperorality. Due to the complete lack of response to treatment and rapid progression, a neurology consultation was made. Investigations included a magnetic resonance of the brain (MRI), electroencephalogram (EEG), autoimmune encephalitis testing, paraneoplastic antibodies, whole body and brain 18F‐fluorodeoxyglucose positron emission tomography (FDG‐PET), screening for late‐onset metabolic diseases and genetic testing.

**Result:**

The MRI of the brain revealed clear‐cut bilateral frontal and temporal atrophy, PET‐FDG showed severe hypometabolism in the same areas. All other investigations were negative. In the following months, behavioral symptoms worsen despite treatment. Ten courses of electroconvulsive therapy were prescribed as a rescue treatment, with some improvement. Although a primary psychiatric condition was considered, imaging findings suggested a neurodegenerative disease. After a wide investigation to exclude other conditions, bvFTD was diagnosed.

**Conclusion:**

Very early onset behavioral variant frontotemporal dementia (bvFTD) is an uncommon clinical presentation of frontotemporal dementia (FTD). A review of the literature identified 18 patients with an onset age of 25 years or younger, with the youngest patient reported to be 14 years old. Due to its early onset and primarily neuropsychiatric symptoms, bvFTD is often misdiagnosed as a psychiatric condition. In these cases, the most commonly reported pathology is tau protein, while mutations are frequently found in the MAPT gene. This differs from typical FTD, where the most commonly reported pathology is TDP‐43 protein. Although rare, it is important to consider bvFTD even in very young patients presenting with new, significant behavioral disturbances.

